# Tinea Capitis Infection in School Children of Nepal

**DOI:** 10.2188/jea.11.126

**Published:** 2007-11-30

**Authors:** Sangeeta Baral Basnet, Narayan Bahadur Basnet, Masataro Hiruma

**Affiliations:** 1 Department of Epidemiology and Environmental Health, Juntendo University School of Medicine.; 2 Department of Pediatrics, The University of Tokyo.; 3 Department of Dermatology, Juntendo University School of Medicine.

**Keywords:** Nepal, risk factors, school children, tinea capitis, *Trichophyton violaceum*

## Abstract

From among 428 Nepalese schoolchildren hair samples of 102 children with clinical features of tinea capitis, obtained by the sterile hairbrush method, were examined by mycological techniques.

Age varied between 4-16 years. Itching was experienced by 96.1% sample subjects and hair loss by 32.4%. Of the 102, 11 (10.8%) were positive for Trichophyton violaceum (TV), 6 being from urban areas, the rest from rural areas. Amongst the 11 patients, 7 (63.6%) were girls and rest boys. Statistical associations were observed between the place of haircut and isolation of the organism (X2 = 15.2, p <0.01). Statistical association was also present between frequency of bathing and isolation of organism. Sharing of combs was associated with the culture-positive subjects. The prevalence of tinea capitis in the urban and rural children was 2.3% and 3.0%, respectively.

The only isolated organism was TV. An association of the isolation of TV was found with risk factors such as family members, sharing of combs, frequency of bathing with the organism. Hair loss was more common in the urban children. Discouragement of sharing combs, increased frequency of hair washing, and use of uncontaminated hair cutting instruments are recommended.

## BACKGROUND

Our preliminary study conducted in 1997-1998 among 11 Nepali school children in Kathmandu indicated that the common causative organism of tinea capitis was *Trichophyton violaceum* (TV)^1^^)^. It is universally accepted that tinea capitis - which in layman’s term is fungal infection of the scalp, is most common fungal infection in children^2^^, ^^3^^)^. This disease is highly contagious by direct contact with the infected person or from infected fomites^4^^)^.

Various studies have been conducted in several countries clarify epidemiological factors of tinea capitis infection^5^^-^^12^^)^. No such study was conducted on this problem in children in such a geographically and climatically diverse country as Nepal^1^^)^. The objectives of the present study were to detect etiological agents of tinea capitis and its risk factors in school children of Nepal. It also aimed to recommend major preventive measures of tinea capitis in school children.

## SUBJECTS AND METHODS

Two integrated primary-junior high schools, in the Kathmandu area one rural and one urban, were selected after visiting and observing a number of schools at various locations. They were selected to reflect rural-urban difference as well as different habitats and ethnicity. We conducted the study in September 1998. Consent was obtained from the school authorities, children and the guardians of all children prior to the study. The survey procedure involved clinical examination of the scalp of the children and collection of hair samples from the scalp. First, clinical inspection of the scalp of all the present school children was performed. Children with clinical features of tinea capitis (itching, scalp hair loss, scaling in the scalp with or without any characteristic lesion, lesion with discharge, lesion with pain) and other associated scalp findings were recorded. The samples of the hair of those who had tinea capitis scalp lesions as well as those who had no lesions but had clinical features were obtained through the sterile plastic hairbrush method. In all subjects, precise details were obtained by a questionnaire.

The urban school had a total population of 318 enrolled students whereas the rural school had a total population of 238 enrolled students. As the first stage of screening, a total of 263 students were examined clinically in the urban school and a total of 165 students in the rural school. Fifty-five students from the urban school and 73 from the rural were absent on the day of the examination and thus could not be included in the study. The age of the students in both the schools ranged between 4-16 years.

From the two schools, a total of 102 hair samples were extracted from the student population, by the previously reported hair brush technique^1^^)^, 53 from the urban school, and 49 from the rural school. Within three weeks of collecting the samples they were transferred to Tokyo under sterile conditions. These hair brushes were incubated in Sabourads’ Dextrose media and stored at 26°C temperature while being observed, studied and photographed at regular intervals for a period of three months until a diagnosis was obtained.

Data on age, sex, family size, personal habits, hygiene and other related factors were analyzed. Statistical analysis of the data was performed. Mean and percentage distribution were estimated, statistical analysis done by the chi-square test. A p-value of less than 0.05 was considered to indicate a statistically significant difference.

## RESULTS

Of 428 children, 102 had clinical features suggestive of tinea capitis (Table 1) however the organism was isolated in 11 cases. The prevalence of tinea capitis infection confirmed both by clinical features and by hairbrush culture in the child population was 2.6%; 2.3% in urban and 3.0 % rural population. The mean age of the 102 sampled children was 9 years 9 months. There was no urban-rural difference in mean age. The number of family members and siblings and also the family set up did not vary so much in the two set ups. Nine out of the 11 total positive subjects were under the age of 12 years (Table 2). Among the 6 positive cases in the urban school 3 were boys and 3 girls; while in the rural school there were 4 girls and 1 boy. Out of the total of 11 positive cases 7 (63.6%) were girls and 4 (36.4%) were boys. The male to female gender ratio was 1.3.

**Table 1.** Common clinical features of studied subjects by hair brush method (n=102).

Clinical features	Urban Children with clinical features (%)		Rural Children with clinical features (%)		Total Children with clinical features (%)	
Itching	50	(94.3)	48	(98.0)	98	(96.1)
Hair loss	28	(52.8)	5	(10.2)	33	(32.4)
Scaling	37	(69.8)	46	(93.9)	83	(81.4)
Lesion with discharge	4	(7.5)	7	(14.3)	11	(10.8)
Lesion with pain	0	(0)	7	(14.3)	7	(6.9)

**Table 2.** Age wise distribution of studied children and isolation of *Trichophyton violaceum* in two schools of Nepal.

Location	Total students	Age distribution of total subjects			TV isolated/ Clinical features	% of TV isolation
		4-8 yr	9-12 yr	13-16 yr		
Urban	263	4 (22)	2 (14)	0 (17)	6/53	11.3
Rural	165	1 (25)	2 (11)	2 (13)	5/49	10.2
Total	428	5 (47)	4 (25)	2 (30)	11/102	10.8

Numbers in the brackets ( ) indicate children with clinical features.

TV, Trichophyton violaceum; TV positive, isolation of TV; ‘Clinical features’ means presence of any two clinical features like itching, scalp hair loss, scaling in the scalp, lesion with discharge, lesion with pain; and /or characteristic lesion with or without any symptoms.

*Trichophyton violaceum* was isolated in a total of 11 children. Out of this total, 54.5% were from urban and 45.5% from rural schools. Itching, scaling and hair loss were observed in 96.1%, 81.4%, and 32.4%, respectively (Table 1). Itching and hair loss were significantly associated (X2=11.6, p<0.01; X2=12.9, p <0.01, respectively) with the isolation of the organism whereas scaling was not (X2=2.9, NS). Out of the total hair samples obtained there were 33 (32.4%) hair loss cases. Among the 53 urban children, 28 (52.8%) had hair loss. In the 49 rural samples 5 (10.2%) had hair loss. Among the 11 TV positive subjects, 4 children (36.4%) had hair loss.

**Figure 1.**
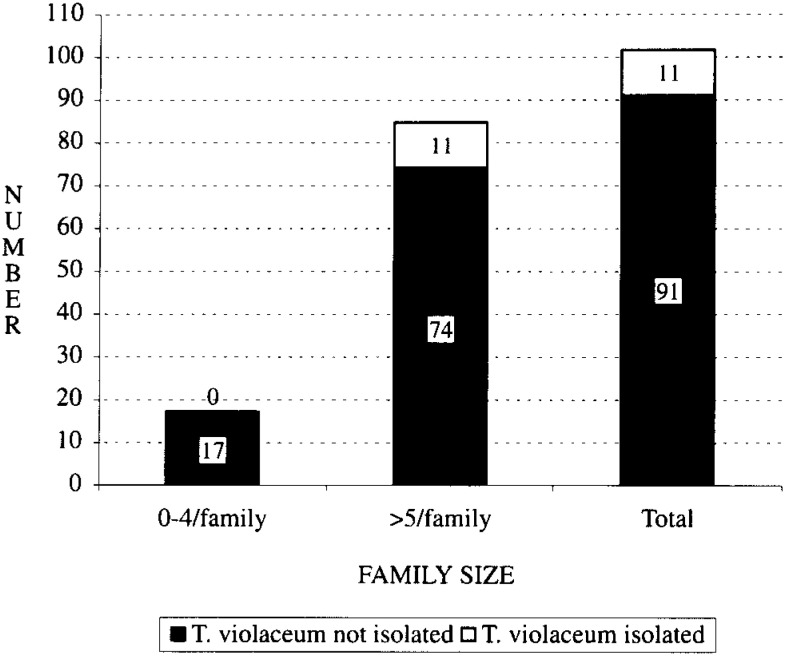
The bar diagram showing the family size and the isolation of Trichophyton violaceum in studied subjects (n=102).

All the positive patients had more than 5 family members and larger families were strongly related with the presence of disease (Figure 1). Mothers were predominantly housewives in both the urban and rural settings. None of the urban mothers were farmers however 10 of the rural mothers were farmers by occupation. There was no association between occupation of the mother, sleep habits and isolation of the organism amongst the 102 children. Sleep habits and habitats were altogether similar in both the areas.

**Figure 2.**
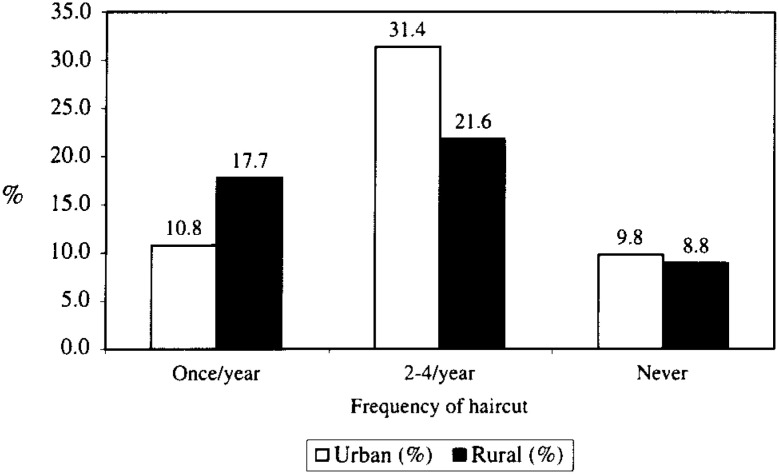
The percentage distribution of urban and rural children in relation to the frequency of haircuts in one year (n=102).

In Nepal there is the traditional habit of using caps by males. None of the positive cases in our study used cap in their daily lives. Though the frequency of haircuts had a similar pattern (Figure 2) a barbershop was the venue of haircuts for 71.1% of the urban children; while 71.4% of the rural population had haircuts at home. Statistical association was observed significantly between the place of haircut, and isolation of the organism (X2=15.2, p <0.01) (Table 3). 100% of the urban school children combed their hair every day, however only 81.6% combed daily among rural school children. Among the urban children who combed daily, 83.0% used a common comb among family members. Among the rural children, 59.2% shared the comb within the family. The sharing of comb was significantly associated with the culture positive cases associated (X2 = 11.9, p <0.01).

**Table 3.** Table showing the association of risk factor and isolation of *Trichophyton violaceum* in total examined subjects (n=102).

Risk factor		TV Positive	TV Negative	Total
Place of haircut	Barber	6	38	44
	Home	4	39	43
	Total	10	77	87
Sharing of comb	Yes	9	68	77
	No	2	23	25
	Total	11	91	102
Frequency of bathing	>3 times/week	3	19	22
	<3 times/week	8	72	80
	Total	11	91	102

TV, *Trichophyton violaceum*.

Statistical association was observed between frequency (more or less than 3 times per week) of bathing and isolation (X2=20.1, p <0.01). Tap water was the source of water for hair wash in 71.7% of the urban and 51.9% of the rural children. While 19.2% of the rural children used pond water for bathing purposes, pond water was not used at all by the urban school population. There was awareness of hair cleansing in 98.1% and 81.6% of urban and rural school children, respectively.

**Figure 3.**
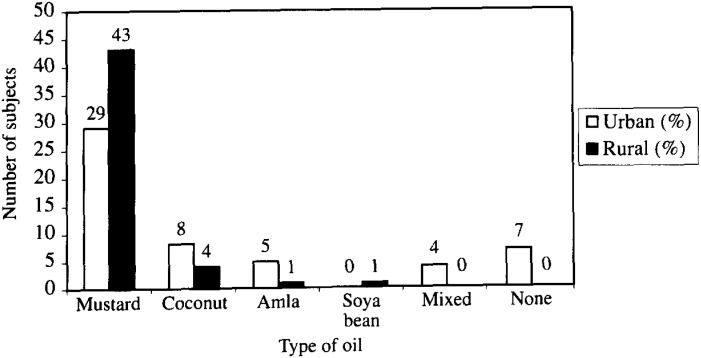
Frequency distribution of types of hair oil used by the studied population (n=102).

Seven children out of the 102 did not use hair oil (Figure 3). Mustard oil was used by 54.7% of the urban children and 87.8% of the rural child population.

## DISCUSSION

Tinea capitis infection is one of the common human dermatophytic infections^5^^)^. This was the first epidemiological study of tinea capitis in Nepalese school children based on the hair brush technique^1^^, ^^13^^)^. One-fifth of the urban and a little less than one-third of rural school children population had clinical symptoms of tinea capitis infection. The causative organism of the 11 total positive subjects was Trichophyton violaceum. We found a higher prevalence of the disease in rural school children^14^^)^. TV was the only organism isolated in this study and in the previous study conducted in 1997-98^1^^)^. No other cause than TV of tinea capitis infection was identified. Children lacked personal hygiene, sanitation and therefore had itching, hair loss, and scaling. These features are common both to tinea capitis infection as well as poor hygiene.

A higher number of positive isolation of the organism in girls than in boys was observed, especially in rural girls, probably due to the tradition of females keeping long hair^1^^)^. Nine out of eleven mothers of the positive subjects were housewives. A previous study in Nigeria showed a significant difference in the rate of infection between male and female schoolchildren as well as between children from different socioeconomic backgrounds^15^^)^.

In the present study, mostly children aged between 4-12 were affected, which was similar to the findings of a large population-based study^12^^)^, although occurrence of infection in adults has also been reported not to be rare^16^^)^.

In our study, all the TV positive children were living in households with more than 5 members, suggesting an association between tinea capitis and large families, but a previous study showed no relationship between overcrowding and scalp infection^9^^)^. We found that sharing of combs was associated with tinea capitis infection whereas previous univariate and multivariate analysis showed no association of carrier state to age, sex, or comb sharing^7^^)^. Sleep habits and habitats were altogether similar in both the urban and rural population. No statistical association was observed between sleep habit and isolation of the organism^7^^)^. This study also revealed that infrequent number of hair washing is one of the risk factors of tinea capitis infection among children.

Symptoms such as itching, scaling and hair loss was common in both the school children population. Hair loss was markedly more in the urban sample population than the rural. It is interesting to note that rural children used more mustard oil whereas the urban population used other varieties of oil together with mustard oil.

Use of mustard oil for hair and scalp massage is common from infancy to elderly in the Nepalese community. A study on the use of hair oil which is commonly used in neighboring India suggests that the use of hair oil had prophylactic effect against tinea capitis. The result of our study was in line with that report. For effective treatment of tinea capitis modern medical treatment is essential^17^^, ^^18^^)^.

In our study, TV was the isolated causative agent of tinea capitis infection in Nepalese children. Some studies in other countries have showed it to be the common causative agent both in children and in adults^14^^, ^^19^^)^. Yet in some other studies TV has also been known to be common in immigrant populations^20^^, ^^21^^)^. However, TV the only causative agent that was identified in this and the previous study^1^^)^ showed that the children of all the studied schools were from a non-immigrant population.

Knowledge of personal hygiene in both urban and rural schools was present but lacking in real life. On the other hand socioeconomic characteristic conditions of the two schools was similar in certain aspects. Improvement in environmental and personal hygiene has been reported to help to decrease infectious diseases including tinea capitis^22^^)^.

The clinical classification of patients before taking hair samples from a large group of population is important. Also explanation of the procedure to the students and teachers is essential for a reproducible study. Before the conduction, the importance of this study was highlighted for everyone involved. Future studies on the geographic and ethnic variations of epidemiological risk factors in Nepal will prove to be useful for both prophylactic and academic purposes.

## CONCLUSION

The only isolated organism from both urban and rural schools was *Trichophyton violaceum*. An association of the isolation of *Trichophyton violaceum* was found with the total number of family members, comb sharing, and frequency of bathing in the Nepalese school children. Hair loss was more common in urban than rural children. Family education on care of hair, increased frequency of bathing, use of uncontaminated instruments for hair cutting and early medical consultation is recommended.

## References

[1] Basnet SB, Basnet NB, Hiruma M, Inaba Y, Makimura K, Kawada A. Mycological examination of the hair samples of 11 school-going Nepalese children suspected of tinea capitis. Mycoses, 2000;43:51-54. 10.1046/j.1439-0507.2000.00541.x 10838847

[2] Elewski B. Tinea capitis. Dermatol Clin, 1996;14:23-31. 8821154 10.1016/s0733-8635(05)70321-1

[3] Ghorpade A, Ramanan C. Tinea capitis and corporis due to *Trichophyton violaceum* in a six-day-old infant. Int J Dermatol, 1994;33:219-220. 8169031

[4] Odom R. Pathophysiology of dermatophyte infection. J Am Acad Dermtol, 1993;28(5 Pt 1):S2-S7. 10.1016/s0190-9622(09)80300-9 8496407

[5] Elewski BE. Tinea capitis: a current perspective. J Am Acad Dermatol, 2000;42(1 Pt 1):1-20;quiz 21-4. 10.1016/s0190-9622(00)90001-x 10607315

[6] Romano C. Tinea capitis in Siena, Italy. An 18-year survey. Mycoses, 1999;42:559-562. 10592701 10.1046/j.1439-0507.1999.00495.x

[7] Pomeranz AJ, Sabnis SS, McGrath GJ, Esterly NB. Asymptomatic dermatophyte carriers in the households of children with tinea capitis. Arch Pediatr Adolesc Med, 1999;153:483-486. 10323628 10.1001/archpedi.153.5.483

[8] Timen A, Bovee L, Leentvaar-Kuijpers A, Peerbooms PJ, Coutinho RA. Tinea capitis in primary school age children in southeastern Amsterdam: primarily due to *Trchophyton tonsurans*. Ned Tijdschr Geneeskd, 1999;143:24-27 [Article in Dutch] 10086094

[9] Figueroa JI, Hawranek T, Abraha A, Hay RJ. Tinea capitis in south-western Ethiopia: a study of risk factors for infection and carriage. Int J Dermatol, 1997;36:661-666. 9352406 10.1046/j.1365-4362.1997.00236.x

[10] Hay RJ, Clayton YM, De Silva N, Midgley G, Rossor E. Tinea capitis in south-east London-a new pattern of infection with public health implications. Br J Dermatol, 1996;135:955-958. 8977718 10.1046/j.1365-2133.1996.d01-1101.x

[11] Laude TA. Skin disorders in black children. Curr Opin Pediatr, 1996;8:381-385. 8954271 10.1097/00008480-199608000-00014

[12] Lobato MN, Vugia DJ, Frieden IJ. Tinea capitis in California children: a population-based study of a growing epidemic. Pediatrics, 1997;99:551-554. 9093297 10.1542/peds.99.4.551

[13] Hubbard TW, de Triquet JM. Brush-culture method for diagnosing tinea capitis. Pediatrics, 1992;90:416-418. 1518699

[14] Ali-Shtayeh MS, Arda HM, Abu-Ghdeib SI. Epidemiological study of tinea capitis in school children in the Nablus area (West Bank). Mycoses, 1998;41:243-248. 9715641 10.1111/j.1439-0507.1998.tb00332.x

[15] Enweani IB, Ozan CC, Agbonlahor DE, Ndip RN. Dermatophytosis in school children in Ekpoma, Nigeria. Mycoses, 1996;39:303-305. 9009650 10.1111/j.1439-0507.1996.tb00143.x

[16] Aste N, Pau M, Biggio P. Tinea capitis in adults. Mycoses, 1996;39:299-301. 9009649 10.1111/j.1439-0507.1996.tb00142.x

[17] Garg AP, Muller J. Inhibition of growth of dermatophytes by Indian hair oils. Mycoses, 1992;35:363-369. 1302812 10.1111/j.1439-0507.1992.tb00895.x

[18] Abdel-Rahman SM, Nahata MC. Treatment of tinea capitis. Ann Pharmacother, 1997;31:338-348. 9066943 10.1177/106002809703100313

[19] Jahangir M, Hussain I, Khurshid K, Haroon TS. A clinico-etiologic correlation in tinea capitis. Int J Dermatol, 1999;38:275-278. 10321943 10.1046/j.1365-4362.1999.00652.x

[20] Maslen MM, Andrew PJ. Tinea due to *Trichophyton violaceum* in Victoria, Australia. Australas J Dermatol, 1997;38:124-128. 9293657 10.1111/j.1440-0960.1997.tb01127.x

[21] Cuetara MS, Del Palacio A, Pereiro M, Noriega AR. Prevalence of undetected tinea capitis in a prospective school survey in Madrid: emergence of new causative fungi. Br J Dermatol, 1998;138:658-660. 9640375 10.1046/j.1365-2133.1998.02181.x

[22] Soda K, Kamakura M, Kitamura K. Research activities of epidemiology in Japan: Infectious disease: fight against infectious diseases. J Epidemiol, 1996;6(Suppl):S61-S66. 8800275 10.2188/jea.6.3sup_61

